# Multi-Bit Resistive Random-Access Memory Based on Two-Dimensional MoO_3_ Layers

**DOI:** 10.3390/nano15131033

**Published:** 2025-07-03

**Authors:** Kai Liu, Wengui Jiang, Liang Zhou, Yinkang Zhou, Minghui Hu, Yuchen Geng, Yiyuan Zhang, Yi Qiao, Rongming Wang, Yinghui Sun

**Affiliations:** 1 Beijing Key Laboratory for Magneto-Photoelectrical Composite and Interface Science, School of Mathematics and Physics, University of Science and Technology Beijing, Beijing 100083, China; m202210756@xs.ustb.edu.cn (K.L.); jiangwengui478@gmail.com (W.J.); b20200364@xs.ustb.edu.cn (L.Z.); m202210796@xs.ustb.edu.cn (Y.Z.); m202310758@xs.ustb.edu.cn (M.H.); m202210741@xs.ustb.edu.cn (Y.G.); yiyuanzhang@xs.ustb.edu.cn (Y.Z.); 2 The State Key Laboratory for Advanced Metals and Materials, University of Science and Technology Beijing, Beijing 100083, China; qiaoyi@ustb.edu.cn

**Keywords:** *α*-MoO_3_ nanosheet, two-dimensional metal oxides, resistive switching layer, resistive random access memory, multilevel storage

## Abstract

Two-dimensional (2D) material-based resistive random-access memory (RRAM) has emerged as a promising solution for neuromorphic computing and computing-in-memory architectures. Compared to conventional metal-oxide-based RRAM, the novel 2D material-based RRAM devices demonstrate lower power consumption, higher integration density, and reduced performance variability, benefiting from their atomic-scale thickness and ultra-flat surfaces. Remarkably, 2D layered metal oxides retain these advantages while preserving the merits of traditional metal oxides, including their low cost and high environmental stability. Through a multi-step dry transfer process, we fabricated a Pd-MoO_3_-Ag RRAM device featuring 2D α-MoO_3_ as the resistive switching layer, with Pd and Ag serving as inert and active electrodes, respectively. Resistive switching tests revealed an excellent operational stability, low write voltage (~0.5 V), high switching ratio (>10^6^), and multi-bit storage capability (≥3 bits). Nevertheless, the device exhibited a limited retention time (~2000 s). To overcome this limitation, we developed a Gr-MoO_3_-Ag heterostructure by substituting the Pd electrode with graphene (Gr). This modification achieved a fivefold improvement in the retention time (>10^4^ s). These findings demonstrate that by controlling the type and thickness of 2D materials and resistive switching layers, RRAM devices with both high On/Off ratios and long-term data retention may be developed.

## 1. Introduction

In recent years, artificial intelligence (AI), centered on machine learning (ML), has advanced rapidly, demonstrating its significant potential in fields such as autonomous driving, natural language processing, and image recognition [1]. This progress has imposed higher demands on data storage and logic operations. High-density, non-volatile memory has emerged as a key enabler for massive data processing in the era of big data. Among next-generation memory technologies, resistive random-access memory (RRAM) stands out as a highly promising candidate, attracting extensive research interest [2,3,4,5]. The basic structure of RRAM typically adopts a metal-insulator-metal (MIM) configuration. This simple architecture not only offers fabrication simplicity and compatibility with existing semiconductor processes but also exhibits excellent scalability for large-scale integration. Furthermore, RRAM’s multilevel cell (MLC) capability supports high-speed read/write operations [6,7], significantly enhancing its storage density [8,9] while reducing its power consumption [10,11]. These characteristics make RRAM particularly well-suited for big data and AI applications.

Conventional RRAM devices primarily utilize metal oxides (e.g., HfO_x_ [12,13,14], TaO_x_ [15,16,17], and TiO_x_ [18,19,20]) as the resistive switching layer. Although metal-oxide-based memristors show great promise for neuromorphic computing and AI applications, their performance and integration levels still fall short of practical requirements. These devices typically suffer from high switching voltages, resulting in elevated power consumption. Moreover, as RRAM devices continue to scale down to achieve more complex functionalities, defects in the metal oxide layer become progressively difficult to control. Such local inhomogeneities and random defects critically impact the switching stability [21,22].

Recently, RRAM devices based on 2D layered materials have demonstrated significant potential in AI and in-memory computing applications [23]. Benefiting from their atomic-scale thickness and atomically smooth surfaces [24], these novel 2D material-based RRAM devices outperform their conventional metal-oxide-based counterparts by achieving lower power consumption, higher integration density, and reduced performance variability [25,26]. When 2D layered metal oxides are employed as the resistive switching layer, such devices not only retain the advantages of traditional metal-oxide RRAM (e.g., high environmental stability) but also enable new performance enhancements. Specifically, α-MoO_3_, with its wide bandgap (>3 eV), exhibits excellent insulating properties, making it an ideal candidate for resistive switching layers to ensure low off-state power consumption. In this study, α-MoO_3_ crystals were synthesized via physical vapor deposition (PVD) followed by mechanical exfoliation to obtain reproducible 2D α-MoO_3_ nanosheets. Through all-transfer, we fabricated Pd-MoO_3_-Ag devices demonstrating a low write voltage (~0.5 V), high switching ratio (>10^6^), and multi-bit storage capability (≥3 bits). By replacing the palladium (Pd) electrode with 2D graphene (Gr) to construct Gr-MoO_3_-Ag devices, an extended retention time was achieved while maintaining the multi-bit storage capability. This work not only provides valuable insights into developing RRAM using 2D layered metal oxides as resistive switching layers but also establishes a universal methodology for characterizing resistive switching materials before and after the forming process.

## 2. Methods

### 2.1. Preparation of α-MoO_3_ Crystals and Thin Flakes

The α-MoO_3_ single crystals were synthesized via physical vapor deposition (PVD) under atmospheric pressure. The synthesis process involved the following: (1) placing a quartz boat containing MoO_3_ powder (Aladdin, 99.95%) at the central heating zone of a tube furnace, with the other quartz boat positioned at the cold zone (25 cm apart) to collect the synthesized crystals; (2) introducing an oxygen carrier gas at a constant flow of 50 sccm to transport the vaporized MoO_3_; (3) heating the furnace to 750 °C at a rate of 20 °C/min and maintaining for 60 min. After growth, the heating was stopped, and the furnace was immediately opened for rapid cooling, and the bulk α-MoO_3_ crystals were collected. For thin flake preparation, multilayer α-MoO_3_ flakes were mechanically exfoliated from the bulk crystals onto polydimethylsiloxane (PDMS) substrates using scotch tape for subsequent dry transfer processes.

### 2.2. Fabrication of Pd-MoO_3_-Ag Devices

First, few-layer α-MoO_3_ flakes were mechanically exfoliated onto a PDMS substrate, then dry transferred onto a SiO_2_/Si wafer with pre-patterned Pd electrodes (50 nm thickness, deposited by thermal evaporation) to establish the Pd-MoO_3_ interfacial contact. The few-layer α-MoO_3_ flakes were then treated with air plasma for 15 s and covered with Ag/Au electrodes using the PDMS-mediated dry transfer. Finally, the Pd-MoO_3_-Ag device was annealed at 200 °C for 1 h in a vacuum to optimize the interfacial contacts.

### 2.3. Fabrication of Gr-MoO_3_-Ag Devices

First, few-layer graphene was mechanically exfoliated onto a PDMS substrate, followed by being dry transferred onto a SiO_2_/Si wafer with pre-patterned, 50 nm thick Pd electrodes to establish the graphene-Pd contact at one end to prevent direct contact between the probe and the graphene and to avoid damaging the graphene. Then, few-layer α-MoO_3_ flakes were mechanically exfoliated onto another PDMS substrate and, subsequently, dry transferred onto the surface of the graphene on the SiO_2_/Si wafer, creating an α-MoO_3_-graphene contact at the other end of the graphene. The upper surface of the few-layer α-MoO_3_ flake was treated with air plasma for 15 s. Then, Ag/Au electrodes were dry transferred onto the α-MoO_3_ surface by PDMS mediation. Finally, the Gr-MoO_3_-Ag device was annealed at 200 °C for 1 h in a vacuum to optimize the interfacial contacts.

### 2.4. Fabrication of Detachable Devices

The fabrication process of the detachable device involved the following: first, a polyvinyl alcohol (PVA, average polymerization degree-2688) layer was spin-coated onto a SiO_2_/Si substrate, followed by the dry transfer assembly of a Gr-MoO_3_ heterostructure, where multilayer graphene served as the bottom electrode under the MoO_3_ flake. Secondly, Ag/Au electrodes were transferred onto another SiO_2_/Si substrate and spin-coated with another kind of PVA (average polymerization degree-0588). The PVA-0588 layer was precisely cut off around the electrodes, and a PDMS substrate was used to tear off the cut PVA and the Ag/Au electrodes together. Finally, we aligned and transferred the Ag/Au electrodes embedded in the PVA onto the pre-formed Gr-MoO_3_ heterostructure to obtain the Gr-MoO_3_-Ag stacked device.

Thin films were fabricated using PVAs with average polymerization degrees of 2688 and 0588. DP-2688: 8 wt% of solution was pipetted onto substrates, followed by two-step spin-coating (500 rpm for 5 s and 8000 rpm for 60 s). DP-0588: 5 wt% of solution was pipetted and spin-coated identically, except for the 4500 rpm for the 60 s for high-speed step. All the films were cured at 70 °C for 60 s on a hot plate.

### 2.5. Characterizations

X-ray diffraction (XRD) measurements were conducted on an X-ray diffractometer (Model: Rigaku SmartLab (3); Beijing, China) using Cu K*_α_* radiation (*λ* = 0.154056 nm) under the ambient conditions. The X-ray photoelectron spectroscopy (XPS) analysis was carried out on an X-ray energy spectrometer (Model: Thermo Scientific K-Alpha; Beijing, China) employing monochromatic Al K*_α_* X-ray with an operating spot of 400 μm in diameter. The binding energy was calibrated to the adventitious carbon C 1s peak at 284.80 eV. Raman spectroscopy was performed at room temperature using a confocal Raman system (Model: Horiba Jobin Yvon HR800; Beijing, China) with a 532 nm excitation laser focused through a 100×/N.A. 0.90 objective to achieve a spot size of approximately 1.5 μm, providing a spectral resolution of 1.5 cm^−1^. The surface topography and the thickness of the samples were characterized by atomic force microscopy (Model: Bruker Multimode 8; Beijing, China). A focused ion beam (Model: Thermo Scientific Helios 5 UX DualBeam; Beijing, China) was utilized to prepare the cross-sectional sample of the Pd-MoO_3_-Ag device. Transmission electron microscopy (Model: Titan ETEM G2; Beijing, China) was used to characterize the microstructure of the α-MoO_3_ flake and the Pd-MoO_3_-Ag heterostructure.

## 3. Results and Discussion

### 3.1. Characterizations of α-MoO_3_

**Figure 1:**
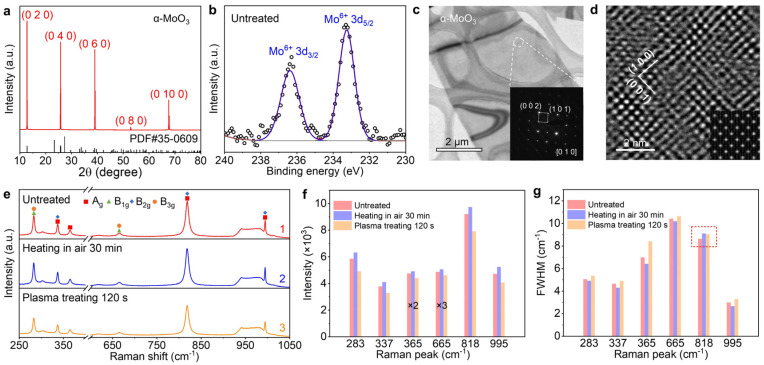
Structural and chemical state characterizations of α-MoO_3_ flakes. (**a**) The XRD pattern of the α-MoO_3_ flakes. (**b**) The high-resolution XPS spectrum and the corresponding peak fitting for Mo 3d core levels in α-MoO_3_. (**c**,**d**) Low- and high-resolution TEM images of the α-MoO_3_ flake. The inset in (**c**) shows the selected-area electron diffraction (SAED) pattern from the region marked by the white, dashed circle. The inset in (**d**) displays the simulated HRTEM image in the [0 1 0] zone axis. (**e**) The Raman spectra of α-MoO_3_ flakes under different treatments: as-prepared, after annealing in air for 30 min at 200 °C, and after air plasma treating for 120 s. (**f**,**g**) A comparison of the peak intensity and the value of the full-width-at-half-maximum (FWHM) for the different Raman vibration modes in (**e**). The peak intensities of the Raman peaks at 365 and 665 cm^−1^ were multiplied by 2 and 3 times for clarity.

α-MoO_3_ is a layered oxide composed of double-layered MoO_6_ octahedra parallel to the (0 1 0) plane, with adjacent layers bonded by relatively weak van der Waals forces. In the MoO_6_ octahedron, each Mo atom is surrounded by six oxygen atoms, and these octahedra connect through shared vertices and edges to form a 2D layered network [27]. X-ray diffraction (XRD) was used to characterize the crystallinity of bulk α-MoO_3_ crystals obtained via the PVD method. As shown in Figure 1a, distinct diffraction peaks appear at 12.8°, 25.7°, 39.0°, 52.8°, and 67.6°, corresponding to the (0 2 0), (0 4 0), (0 6 0), (0 8 0), and (0 10 0) planes of α-MoO_3_ (PDF#35-0609), respectively. The observation of only (0 *k* 0) peaks in the XRD pattern indicates that the [0 1 0] direction of the α-MoO_3_ crystal is perpendicular to the SiO_2_/Si substrate, while the α-MoO_3_ flakes are parallel to it. The diffraction results correspond to the space group *Pbnm* with lattice parameters of a = 0.3962 nm, b = 1.3855 nm, and c = 0.3699 nm. The XRD result reveals that the MoO_3_ bulk that was grown by PVD is a single crystal.

X-ray photoelectron spectroscopy (XPS) was employed to characterize the elemental composition and chemical states of the α-MoO_3_ flakes. The binding energy scale was calibrated by setting the C 1 s peak to 284.80 eV. As shown in Figure 1b, the characteristic doublet peaks of Mo 3d_3/2_ and Mo 3d_5/2_ in α-MoO_3_ were detected at 236.4 and 233.2 eV, respectively, which can be attributed to the Mo^6+^ oxidation state of the α-MoO_3_ phase [28,29,30].

To investigate the microstructure of α-MoO_3_, transmission electron microscopy (TEM) and high-resolution TEM (HRTEM) were utilized. Figure 1c presents a low-magnification TEM image of a typical few-layer α-MoO_3_ flake. The inset in Figure 1c displays the selected-area electron diffraction (SAED) pattern obtained from the region marked by the white, dashed circle, where the diffraction spots corresponding to the (1 0 1) and (0 0 2) planes of α-MoO_3_ are clearly indexed. These results indicate the [0 1 0] zone axis for α-MoO_3_, consistent with the *Pbnm* space group of α-MoO_3_. Figure 1d shows a representative HRTEM image of α-MoO_3_, revealing two sets of perpendicular lattice fringes with measured spacings of 0.39 and 0.37 nm, which match the interplanar distances of the (1 0 0) and (0 0 1) planes of α-MoO_3_ [31]. The inset in Figure 1d reveals a simulated HRTEM image along the [0 1 0] zone axis, demonstrating excellent agreement with the experimental image, thereby providing further confirmation of the α-MoO_3_ phase.

Raman spectroscopy serves as a crucial technique for characterizing crystal structures and phonon vibration modes of materials. The Raman spectrum of α-MoO_3_ exhibits multiple characteristic peaks in the range of 250~1000 cm^−1^, primarily associated with the vibration modes of Mo-O bonds in its crystal structure. As shown in Figure 1e, distinct Raman peaks originate from the different vibration modes of the Mo-O-Mo bridging bonds and two types of terminal oxygen (Mo=O). For the terminal oxygen vibrations, the wagging mode appears at 283 cm^−1^ (B_1g_, B_3g_), the shearing mode at 365 cm^−1^ (A_g_), the symmetric stretching mode at 818 cm^−1^ (A_g_, B_2g_), and the asymmetric one at 995 cm^−1^ (A_g_, B_2g_). For Mo-O-Mo bridging bond vibrations, the bending mode appears at 337 cm^−1^ (A_g_, B_2g_) and the stretching one at 665 cm^−1^ (B_1g_, B_3g_) [32]. We systematically investigated the evolution of the Raman spectra of α-MoO_3_ flakes before and after thermal or surface treatments (Figure 1e). While the peak positions remained nearly unchanged, significant variations in the peak intensity and the full-width-at-half-maximum (FWHM) were observed. Detailed data can be found in Tables S1 and S2 in the Supporting Information. Figure 1f shows that the peak intensity of all the Raman modes is enhanced after annealing in air at 200 °C for 30 min, while a subsequent air plasma treatment for 120 s can reduce the peak intensity, compared with the untreated counterpart. As shown in Figure 1g, the FWHM values of five characteristic Raman peaks (excluding the 818 cm^−1^ peak) decreased after annealing but increased beyond the pre-treatment levels following plasma treatment. It is noted that the change in the FWHM values of the Raman peak at 818 cm^−1^ shows an opposite trend (highlighted by the red, dashed frame in Figure 1g): its FWHM increased after heating but decreased after plasma treatment, though remaining larger than the initial value. In a Raman spectrum, a perfect crystalline lattice produces sharp vibration peaks with high intensity and small FWHM values. Defects (e.g., vacancies, dislocations) or amorphous phases in the materials can give rise to complex phonon modes, frequency shifts, wider peak widths, and weaker intensities. Our analysis suggests that thermal annealing in air helps repair defects in α-MoO_3_, while plasma treatment introduces additional defects. The anomalous behavior of the 818 cm^−1^ peak may stem from the surface reactions with atmospheric O_2_ during heating, which increases the concentration of terminal oxygen in the Mo=O with the enhanced peak intensity. In the subsequent cooling process, airborne molecules may be adsorbed, which perturb the symmetric stretching vibrations of terminal oxygen, thus resulting in larger FWHM values.

The characterization and analysis in Figure 1 demonstrate that the α-MoO_3_ crystals prepared by the PVD method exhibit good overall crystallinity while containing localized oxygen vacancies or other defects. Thermal annealing in air can reduce defect concentration, thereby enhancing the Raman peak intensities of both Mo-O-Mo and Mo=O bonds. Conversely, the plasma treatment introduced additional defects into the α-MoO_3_. This systematic investigation on different treatments provides a simple, yet effective, approach for the controllable modulation of oxygen vacancies or defects in the resistive switching layer of RRAM devices.

### 3.2. Fabrication and Resistive Switching Performance of RRAM

**Figure 2:**
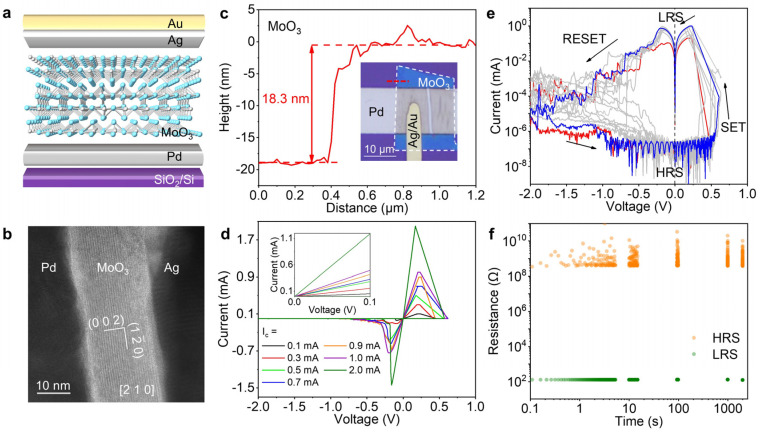
The device architecture, TEM characterization, and resistive switching performance of the Pd-MoO_3_-Ag device. (**a**) A schematic cross-sectional view of the Pd-MoO_3_-Ag device. (**b**) A cross-sectional TEM image showing the layered device configuration. (**c**) The thickness measurement of the α-MoO_3_ switching layer. Inset: the optical micrograph of the device with thickness profiling along the red, dashed line. (**d**) *I*-*V* characteristics under different compliance currents (*I*_c_). The inset image highlights the low-voltage region (0~0.1 V). (**e**) Resistive switching endurance characteristics. The black arrows indicate the scanning voltage sequence of 0 → 1.0 → 0 → −2.0 → 0 V. (**f**) Retention performance evaluation.

The Pd-MoO_3_-Ag device was fabricated with a vertical architecture on a SiO_2_/Si substrate containing pre-patterned Pd electrodes. Figure 2a shows its cross-sectional schematic. Palladium was selected as the inert electrode material due to its excellent stability. Pd electrodes with a thickness of 50 nm were deposited via electron-beam evaporation. The α-MoO_3_ resistive switching layer was prepared by mechanically exfoliating PVD-grown α-MoO_3_ crystals, followed by a PDMS-mediated dry transfer onto the Pd electrode. Atomic force microscopy (AFM) measurements determined the α-MoO_3_ layer thickness to be approximately 18.3 nm (Figure 2c). The α-MoO_3_ surface underwent a 15 s air plasma treatment to introduce controlled defects intentionally. Finally, a patterned Ag/Au electrode was dry transferred onto the α-MoO_3_ flake, with subsequent 1 h vacuum annealing at 200 °C to optimize the electrode interface contact. A high-resolution transmission electron microscopy (HRTEM) analysis of the focused ion beam (FIB)-prepared device cross-section revealed well-defined van der Waals interfaces in the pristine device before the electrical measurements (Figure 2b). The HRTEM image distinctly exhibits lattice fringes in the resistive switching layer, consistent with single-crystalline α-MoO_3_ oriented along the [2 1 0] zone axis. Figure S1b,c display the fast Fourier transform pattern of the cross-section and the simulated electron diffraction pattern along the [2 1 0] zone axis.

Notably, our developed Pd-MoO_3_-Ag devices demonstrate multilevel storage capability through the controlled regulation of the compliance current (*I*_c_). In RRAM devices, the SET process typically involves the formation of conductive filaments. A failure to set a compliance current may induce excessive current flow, promoting the overgrowth of the conductive filaments and potentially causing irreversible device breakdown. The imposition of a compliance current enforces a maximum current limit during SET operation, which safeguards the device from damage. Moreover, varying compliance currents directly modulate the growth extent of conductive filaments, consequently determining the resultant resistance value. As shown in Figure 2d, distinct intermediate resistive states emerge in the current-voltage (*I*-*V*) characteristics under different *I*_c_ values. Figure S2 further reveals that under identical voltage pulse stimulation (1.2 s duration, 1 V amplitude), varying the *I*_c_ settings generates discrete current values at a fixed read voltage of 0.1 V, showing a monotonic increase in the read current with *I*_c_. Figure 2d shows seven distinct current values corresponding to seven *I*_c_ settings at a 0.1 V bias, demonstrating a clear positive correlation between the current magnitude and the *I*_c_. Combined with the high-resistance state (HRS), the device achieves at least eight non-volatile resistive states, enabling a reliable 3-bit storage capacity. This controlled multilevel switching behavior highlights the device’s exceptional multi-bit storage capability through precise compliance current engineering.

The SET operation is the process of switching the RRAM device from a high-resistance state to a low-resistance state. The RESET operation is the process of switching the RRAM device back from a low-resistance state to a high-resistance state.

The resistive switching characteristics of the Pd-MoO_3_-Ag device under the controlled *I*_c_ are systematically investigated in Figure 2e. Cyclic *I*-*V* measurements demonstrate a highly reproducible switching behavior across different *I*_c_ conditions, confirming a good operational stability. In our measurement, a scanning voltage sequence of 0 → 1.0 → 0 → −2.0 → 0 V is used. Because of the limitation of our testing equipment, once the current reaches the compliance value (*I*_c_), the voltage is controlled to regulate the current, thus preventing any further increase in the actual value of the voltage. The device achieves a remarkably low write voltage of ~0.5 V, providing two key advantages: (1) a reduced dynamic power consumption (*P* ∝ *V*^2^), particularly crucial for high-density memory and low-power electronics; (2) a low operational voltage, significantly suppressing Joule heating and mitigating thermally induced performance degradation. As the scaling of the semiconductor technology nodes and the reduction of the core operating voltages continue, such low-voltage RRAM devices demonstrate direct compatibility with advanced process nodes, thereby eliminating the need for additional voltage conversion circuitry.

The device exhibits an erase voltage of −0.7 to −2.0 V, with this modest magnitude providing dual benefits, namely reduced operational energy consumption and suppressed material fatigue (like electrode material diffusion), enhancing cycling endurance. The device exhibits an exceptional switching ratio exceeding 10^6^. This enhanced resistance contrast enables two critical operational advantages: (1) improved noise immunity through robust differentiation between HRS and LRS, significantly enhancing read reliability; (2) a reduced read voltage requirement (≤0.1 V) that enables precise state discrimination while simultaneously reducing power consumption. Figure S3 shows the resistance variation in the HRS and the LRS as a function of the cycle number.

Retention time, a critical performance metric for non-volatile RRAM, quantifies the duration during which stored data can be reliably preserved without power. To evaluate this parameter, we applied a programming voltage pulse (1 s duration, 1 V amplitude) followed by read operations at 0.1 V at progressive time intervals (0, 10, 100, 1000, and 2000 s). As shown in Figure 2f, the Pd-MoO_3_-Ag device demonstrates a retention capability exceeding 2000 s, confirming stable non-volatile storage characteristics, an essential requirement in memory applications. Notably, in the HRS, the current becomes extremely low, approaching the measurement limit of our instrumentation, resulting in inherent signal fluctuations. This instrumentation-limited current measurement (Figure 2e) consequently induces calculated resistance variations in the HRS, as shown in Figure 2f.

**Figure 3:**
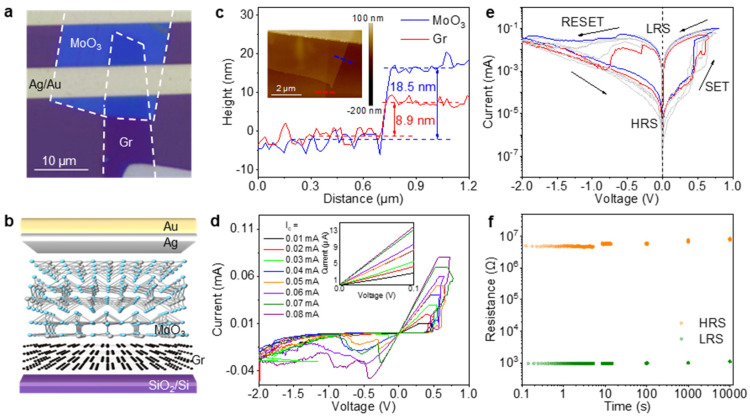
Optical image, structural schematics, and the performance characterization of the Gr-MoO_3_-Ag device. (**a**) A top-view optical image of the Gr-MoO_3_-Ag device. (**b**) A cross-sectional schematic of the heterostructure configuration. (**c**) The thickness measurement of the α-MoO_3_ switching layer and the multilayer Gr electrode. Inset: a zoomed-in AFM topography of the device with thickness profiling along the blue (α-MoO_3_) and red (Gr) dashed lines. (**d**) *I*-*V* characteristics under different *I*_c_s. The inset image highlights the low-voltage region (0~0.1 V). (**e**) Resistive switching endurance characteristics. The black arrows indicate the scanning voltage sequence of 0 → 1.0 → 0 → −2.0 → 0 V. (**f**) Retention time evaluation.

The Pd-MoO_3_-Ag device with a 2D MoO_3_ resistive switching layer demonstrated an excellent stability, a low write voltage, a high switching ratio, and a multi-bit storage capability in previous tests. However, its 2000 s retention time posed limitations for practical applications. Two-dimensional layered materials allow the intercalation of external particles between adjacent layers. When used as electrodes, they can modulate the ion migration dynamics. To enhance the retention characteristics, we developed a Gr-MoO_3_-Ag device through the replacement of the Pd bottom electrode with 2D graphene (Gr) via a similar dry transfer process. The device architecture is illustrated in Figure 3a (top view) and Figure 3b (side view). Graphene exhibits exceptional electrical conductivity, thermal conductivity, and mechanical flexibility [33], while its atomically smooth surface effectively reduces the defect concentration at the MoO_3_ interface. This synergistic combination significantly improves the reliability and stability of the RRAM device.

Figure 3a,b show the optical image and the structural schematics of the Gr-MoO_3_-Ag device. The AFM image in the inset of Figure 3c displays exceptionally smooth material surfaces, where red and blue profile lines correspond to step heights of 8.9 nm (Gr) and 18.5 nm (α-MoO_3_). To compare with the cycling test data of the Pd-electrode device, the same scanning voltage sequence of 0 → 1.0 → 0 → −2.0 → 0 V was applied to this device. As illustrated in Figure 3e, a complete reset can be achieved under a smaller *I*_c_, which is confirmed by the overlapping of the *I*-*V* curves near −2.0 V (red curve). Under a higher *I*_c_, the increased accumulation of conductive filaments in the MoO_3_ layer requires larger negative voltages to successfully finish the RESET operation. Consequently, within the same voltage range, the complete reset cannot be accomplished under a higher *I*_c_. Figure 3d demonstrates *I*-*V* characteristics with multiple intermediate resistive states under different *I*_c_s. The inset of Figure 3d confirms that, including the HRS, the Gr-MoO_3_-Ag device achieves eight distinct resistive states through *I*_c_ modulation, enabling a reliable 3-bit storage capability.

Figure 3e illustrates the resistive switching behavior of the Gr-MoO_3_-Ag device. Cyclic *I*-*V* measurements under different *I*_c_s reveal highly superimposable hysteresis curves, indicating a good cycling stability. Key operational parameters include a write voltage of ~0.5 V, a switching ratio of ~5 × 10^3^, and an erase voltage range of −1.0 to −2.0 V. As shown in Figure 3f, the Gr-MoO_3_-Ag device exhibits a retention time exceeding 10^4^ s. While both the Gr-MoO_3_-Ag and the Pd-MoO_3_-Ag devices enable multi-bit storage capabilities, the comparative analysis demonstrates distinct performance trade-offs: the Pd-MoO_3_-Ag device displayed superior resistive switching characteristics with a higher switching ratio (10^6^ vs. 5 × 10^3^) and higher resistance (10^9^ vs. 5 × 10^6^ Ω). The Pd-electrode devices showed a SET voltage of approximately 0.5 V and a RESET voltage ranging from −0.7 to −2.0 V, while the Gr-electrode devices exhibited a similar SET voltage of ~0.5 V but required a slightly higher RESET voltage window of −1.0 to −2.0 V. Conversely, the Gr-MoO_3_-Ag device achieves markedly extended retention characteristics, maintaining the data integrity for five times longer than the Pd-based device (10,000 vs. 2000 s at room temperature).

### 3.3. Analysis of Device Operating Mechanisms

**Figure 4:**
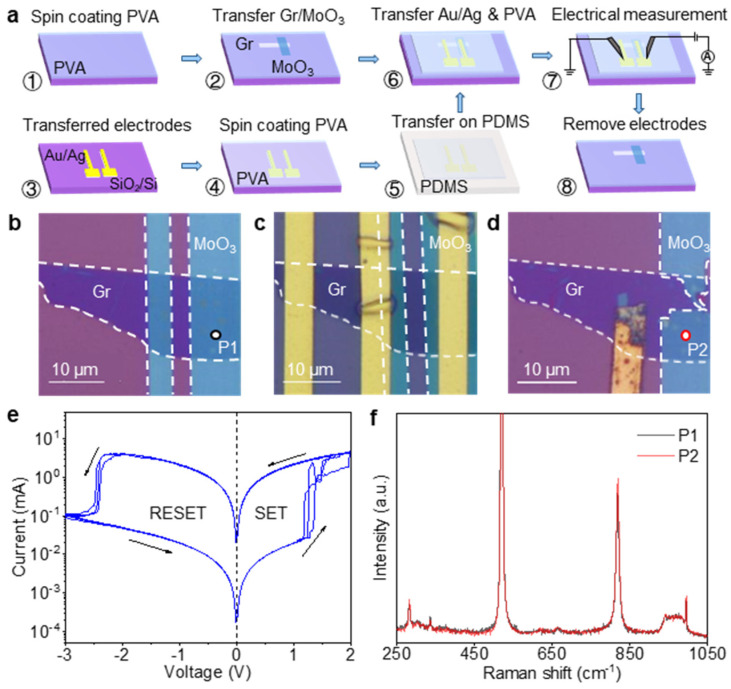
A schematic illustration of the detachable device fabrication approach and the performance characterization. (**a**) The step-by-step assembly/disassembly process (steps ①–⑧). (**b**) An optical micrograph of the initially fabricated Gr-MoO_3_ heterostructure (step ②). (**c**) An optical micrograph of the completed Gr-MoO_3_-Ag device after Ag/Au electrode integration (step ⑥). (**d**) The post-test Gr-MoO_3_ heterostructure after electrode removal (step ⑧) with a residual PVA layer on the substrate. After electrode stripping, the residual Ag/Au electrode and the partial loss of the MoO_3_ layer can be observed. (**e**) The resistive switching performance of the device in (**c**). (**f**) A comparison of the Raman spectra of α-MoO_3_ before (black curve; “P1” in (**b**)) and after (red curve; “P2” in (**d**)) the electrical testing.

To compare the property of the α-MoO_3_ resistive switching layer in RRAM devices before and after electrical *I*-*V* cycling tests, we adopted a reconfigurable device fabrication method [34], with the procedures illustrated in Figure 4a. The procedures include the following steps: (1) Spin-coating a polyvinyl alcohol (PVA, average polymerization degree, 2688) layer on a SiO_2_/Si substrate. (2) Dry transferring it to construct a Gr-MoO_3_ heterostructure on the PVA layer (Figure 4a②). In this configuration, multilayer graphene serves as the bottom electrode beneath the MoO_3_ flake, as shown in the optical image (Figure 4b). (3) Performing the initial Raman characterization of the MoO_3_ layer from P1, as marked in Figure 4b. The result is shown by the black curve in Figure 4f. Subsequent steps involved the following: (4) Transferring pre-patterned Ag/Au electrodes onto another SiO_2_/Si substrate (Figure 4a③). (5) Spin-coating another kind of PVA layer (average polymerization degree, 0588) and patterning it around the electrodes. (6) Delaminating the patterned PVA layer with embedded Ag/Au electrodes using PDMS (Figure 4a⑤). (7) Transferring this stack onto the pre-formed Gr-MoO_3_ heterostructure to complete the assembly of the Gr-MoO_3_-Ag device (Figure 4a⑥), with the final structure shown in Figure 4c. For electrical measurements, probes directly penetrate the PDMS thin layer to contact the Ag/Au electrodes (Figure 4a⑦). In practice, the leftmost and rightmost metal electrodes were connected to graphene and MoO_3_, respectively. (8) After testing, the PVA (average polymerization degree, 0588) layer and the Ag/Au electrodes were delaminated with adhesive tape, revealing the post-cycling MoO_3_ layer, as illustrated in Figure 4a⑧.

In the experiment, we used a scanning voltage sequence of 0 → 2.0 → 0→ −3.0 → 0 V. Our resistive switching characterization on the Gr-MoO_3_-Ag device (Figure 4e) revealed a distinct switching behavior with a write voltage of ~1.3 V and a switching ratio of > 10^2^. To preserve the integrity of the PVA, both the annealing and the plasma treatment were intentionally omitted during device fabrication, resulting in enlarged interfacial gaps between the MoO_3_ and the electrodes with a reduced surface defect density in MoO_3_. These structural characteristics collectively affect the device performance: the expanded gaps decelerate Ag ion migration, while the sparse defects confine the growth of conductive filaments to specific sites, leading to concentrated filament accumulation at these limited defect locations that elevates the off-state current (*I*_off_) and diminishes the switching ratio. Crucially, this spatially constrained growth of the conductive filaments coupled with the moderated switching ratio synergistically enhances the overall stability of the resistive switching operation.

Following the switching characterization, we independently executed a SET operation through a 0 to 2.0 V voltage sweeping to stabilize the device in its LRS prior to mechanical delamination. The PVA (degree of polymerization, 0588) layer and the Ag/Au electrodes were subsequently exfoliated using adhesive tape, thereby exposing the post-cycling MoO_3_ layer. The optical image in Figure 4d shows the residual Ag/Au electrode and the partial loss of the MoO_3_ layer after electrode stripping. Subsequent Raman measurements at the identical location (P2 marked in Figure 4d) demonstrated a remarkable spectral consistency between the post-cycling (red spectrum, Figure 4f) and the pre-test (black spectrum) conditions, with both the peak positions and the relative intensities showing negligible variation. This indicates the preservation of the α-MoO_3_ crystalline integrity following multiple SET/RESET cycles under mild operating conditions. The exceptional structural stability of the 2D α-MoO_3_ not only ensures enhanced device endurance and operational reliability but also positions this material as a superior alternative to conventional oxide-based, non-volatile memory. Figure S4 shows the invariance in both the peak positions and the intensities of the Raman spectra of Gr before and after performance testing.

**Figure 5:**
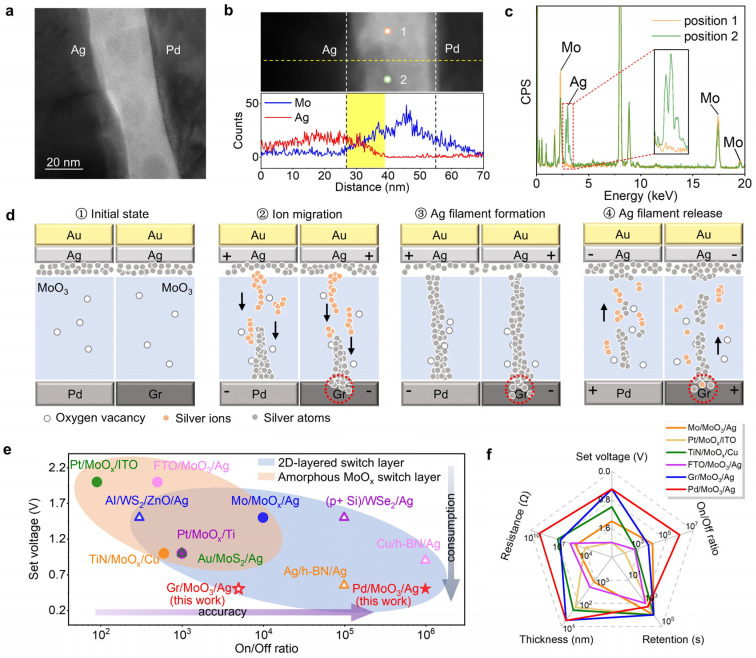
(**a**) A TEM image of the cross-section of the Pd-MoO_3_-Ag device after >100 *I*-*V* cycles and retention tests. (**b**) A line profile of the elemental distribution along the yellow, dashed line in the top panel. (**c**) X-ray energy dispersive spectra (EDS) captured from positions 1 and 2 marked in (**b**). (**d**) A schematic of the working mechanism of the Pd-MoO_3_-Ag and the Gr-MoO_3_-Ag devices during the SET and RESET processes. (**e**) A comparison of the set voltage and the On/Off ratio for different RRAM devices. (**f**) A comparison of the switching layer thickness, resistance in HRS, set voltage, On/Off ratio, and retention time for various molybdenum-oxide-based resistive switching devices.

The working principle of RRAM is primarily based on two physical mechanisms: the valence change mechanism (VCM) and the electrochemical metallization mechanism (ECM) [3,25]. In our experiment, a silver electrode was employed as the active electrode to achieve resistance state switching through the formation and dissolution of conductive channels via silver ion migration. A Pd-MoO_3_-Au device without an active electrode was also prepared as a counterpart. Figure S5 reveals that the intrinsic *α*-MoO_3_ without the Ag electrode exhibits no resistive switching behavior. Therefore, the formation and dissolution of conductive channels of silver ions is indispensable in the MoO_3_-based RRAM. This mechanism is supported by Figure 5a, which shows significant structural changes in the MoO_3_ resistive switching layer after >100 *I*-*V* cycles and retention tests compared to its initial crystalline lattice (Figure 2b). The contrast variations observed in the MoO_3_ layer indicate material modifications during operation. Furthermore, the line scanning of the elemental distribution along the yellow, dashed line in the top panel of Figure 5b revealed the simultaneous detection of Ag and Mo elements within the 27~39 nm range (Figure 5b, bottom panel), confirming the incorporation of Ag into the switching layer. Additionally, the point-scan EDS analysis at positions 1 and 2, marked in Figure 5b (top panel), demonstrated the presence of Ag only at position 2. These results collectively indicate that the silver injection into the MoO_3_ layer occurs through random and localized penetration. This conclusion is further supported by the contrast variations observed in Figure 5b.

The operational mechanisms during the SET and RESET processes in both the Pd-MoO_3_-Ag and the Gr-MoO_3_-Ag RRAM devices, where Pd or graphene serves as the bottom electrode and Ag as the top electrode, are schematically illustrated in Figure 5d. The process begins with the inherent presence of low-concentration oxygen vacancies and other defects in the α-MoO_3_ resistive switching layer, which unavoidably form during oxide preparation (Figure 5d①). In the SET process, as shown in Figure 5d②, when a positive voltage is applied to the active electrode (with the inert electrode negatively biased), Ag atoms at the interface undergo electrochemical oxidation to form Ag ions. These Ag ions then migrate through the resistive switching layer toward the inert electrode under the influence of the electric field. Upon reaching the inert electrode surface, the Ag ions gain electrons and are reduced to Ag atoms. The accumulation of Ag atoms under the bias voltage progressively forms a conductive filament that bridges the two electrodes. Once the conductive filament is fully established, the device switches to the LRS (Figure 5d③). In the RESET process, as shown in Figure 5d④, a positive voltage is applied to the inert electrode (with the active electrode negatively biased), and the Ag atoms in the conductive filament are oxidized into Ag ions. These Ag ions subsequently migrate toward the active electrode, causing the conductive filament to gradually dissolve. When the filament is completely ruptured, the conductive pathway is eliminated, and the device returns to the HRS.

The operational principles of the Pd-MoO_3_-Ag and the Gr-MoO_3_-Ag devices differ subtly. Owing to the unique layered structure of van der Waals materials, their interlayers can accommodate the intercalation of external particles (e.g., atoms, molecules, or ions). When graphene acts as the electrode, silver ions may intercalate into the surface layers of multilayer graphene [35], where they are subsequently reduced to silver atoms, as illustrated in Figure 5d②,③. This intercalation process could hinder the migration rate of silver ions, thereby influencing the accumulation of silver atoms in the resistive switching layer. Consequently, as shown in Figure 3e, the Gr-MoO_3_-Ag device exhibits a significantly increased off-state current due to the accumulation of silver atoms. Because of this, the mechanism can promote the conductive filament’s stability, leading to improved retention characteristics.

As shown in Figure 5e, a comparative analysis of the resistive switching (RS) devices employing either molybdenum oxides or 2D layered materials as resistive switching layers [36,37,38,39,40,41,42,43,44,45] reveals that our devices achieve superior performances in both the write voltage and the On/Off ratio. Furthermore, Figure 5f specifically benchmarks molybdenum-oxide-based RS devices across five key parameters—the switching layer thickness, resistance in HRS, write voltage, On/Off ratio, and retention time [36,37,38,40]—demonstrating four significant advantages of our devices: thinner switching layers (enabling higher integration density), higher HRS resistance, a lower write voltage (reducing the dynamic power consumption following *P* ∝ *V*^2^), and a higher On/Off ratio (enhancing read reliability). These combined improvements (detailed data are presented in Tables S3 and S4) contribute to a low-power operation with minimal storage errors. This comprehensive performance profile underscores their strong potential for application in next-generation RRAM technology. Compared with recently reported multilevel RRAM devices (Table S5 and Figure S6) [46,47,48,49], our fabricated devices demonstrate a comparable multilevel storage capability while exhibiting significantly lower write voltages.

## 4. Conclusions

In summary, we successfully synthesized bulk α-MoO_3_ crystals via the PVD method and obtained 2D α-MoO_3_ flakes through mechanical exfoliation. Comprehensive characterization using XRD, XPS, Raman spectroscopy, and TEM confirmed the high crystalline quality and the structural integrity of the α-MoO_3_. Subsequently, through a multi-step dry transfer process, we fabricated Pd-MoO_3_-Ag RRAM devices employing 2D α-MoO_3_ as the resistive switching layer, with Pd and Ag serving as inert and active electrodes, respectively. The devices demonstrated excellent performance metrics, including remarkable stability, a low write voltage (~0.5 V), a high switching ratio (>10^6^), and a multilevel storage capability (≥3 bits). However, the retention time of ~2000 s posed practical limitations. To address this issue, we developed Gr-MoO_3_-Ag devices by replacing the Pd electrode with multilayer Gr. Although these devices exhibited a slightly compromised write voltage and switching ratio compared to the Pd-MoO_3_-Ag counterparts, the unique layered structure of Gr enabled the intercalation of Ag atoms/ions, resulting in a fivefold improvement in the retention time (>10^4^ s). This work establishes that the strategic selection and structural optimization of electrode materials (considering their type, thickness, and interfacial properties), combined with appropriate resistive switching layers, can obtain RRAM devices capable of simultaneously achieving both high switching ratios and extended retention times.

## Data Availability

The original contributions presented in this study are included in the article/Supplementary Material. Further inquiries can be directed to the corresponding authors.

## Supplementary Materials

The following supporting information can be downloaded at https://www.mdpi.com/article/10.3390/nano15131033/s1.
