# Supplementary material for: Multi-Bit Resistive Random-Access Memory Based on Two-Dimensional MoO3 Layers
Source: Nanomaterials (Basel). 2025 Jul 3;15(13):1033. doi: 10.3390/nano15131033 (PMC12250720; doi:10.3390/nano15131033)
Supplement: Supplementary file 1 [file nanomaterials-15-01033-s001.zip › nanomaterials-3646494-supplementary.pdf]

nanomaterials

Supporting Materials

Multi-bit resistive random-access memory based on two-dimensional MoO_3 layers

Kai Liu [1,#], Wengui Jiang [1,#], Liang Zhou [1], Yinkang Zhou [1], Minghui Hu [1], Yuchen Geng [1], Yiyuan Zhang [1], Yi Qiao [2], Rongming Wang [1,2,*] and Yinghui Sun [1,*]

[1]Beijing Key Laboratory for Magneto-Photoelectrical Composite and Interface Science, School of Mathematics and Physics, University of Science and Technology Beijing, Beijing 100083, China
[2]The State Key Laboratory for Advanced Metals and Materials, University of Science and Technology Beijing, Beijing 100083, China

* Correspondence: to whom correspondence should be addressed: rmwang@ustb.edu.cn and yhsun@ustb.edu.cn

Table S1. Intensity of each Raman peak in Figure 1e.

State \ Raman peak / Intensity	283 cm^{-1}	337 cm^{-1}	365 cm^{-1}	665 cm^{-1}	818 cm^{-1}	995 cm^{-1}
Untreated	5848	3767	2368	1621	9209	4712
Heating in air 30 min	6322	4090	2446	1682	9723	5231
Plasma treating 120 s	4899	3278	2193	1534	7910	4043

Table S2. Values of full width at half maximum (FWHM) of each Raman peak in Figure 1e.

State \ Raman peak / FWHM	283 cm^{-1}	337 cm^{-1}	365 cm^{-1}	665 cm^{-1}	818 cm^{-1}	995 cm^{-1}
Untreated	5.04 cm^{-1}	4.63 cm^{-1}	6.97 cm^{-1}	10.41 cm^{-1}	8.61 cm^{-1}	2.98 cm^{-1}
Heating in air 30 min	4.89 cm^{-1}	4.28 cm^{-1}	6.40 cm^{-1}	10.18 cm^{-1}	9.10 cm^{-1}	2.65 cm^{-1}
Plasma treating 120 s	5.33 cm^{-1}	4.87 cm^{-1}	8.40 cm^{-1}	10.62 cm^{-1}	9.03 cm^{-1}	3.27 cm^{-1}

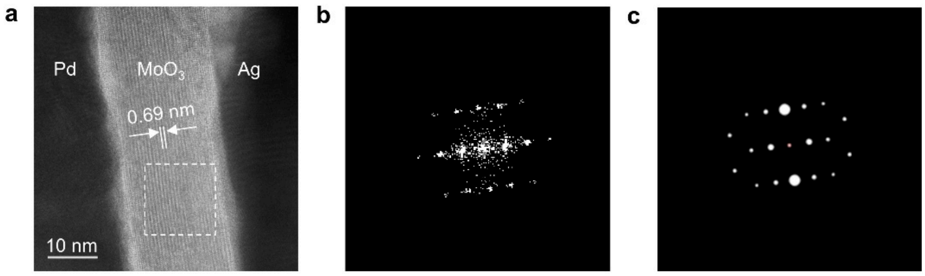

Figure S1. (a) Cross-sectional TEM image of the Pd-MoO_3-Ag device. (b) Fast Fourier transform (FFT) pattern corresponding to the region marked by the white dashed frame in (a). (c) Simulated electron diffraction pattern along the α-MoO_3 [2 1 0] zone axis.

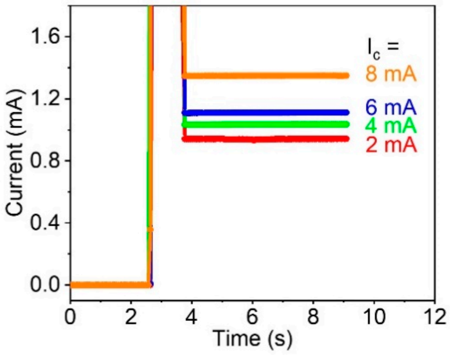

Figure S2. Pulsed test of the Pd-MoO_3-Ag device with a pulse voltage of 1 V and pulse duration of 1.2 s.

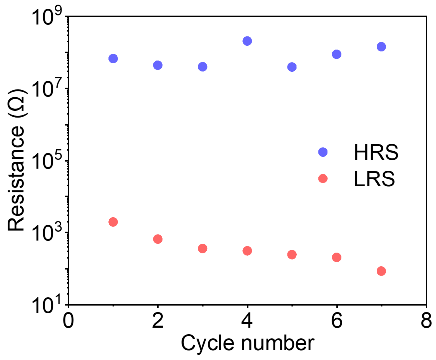

Figure S3. Resistance values of HRS and LRS in Pd-MoO_3-Ag device as a function of cycle number.

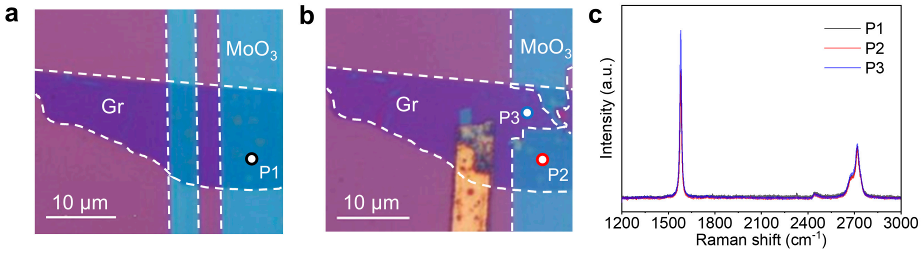

Figure S4. Raman analysis of graphene in detachable Gr-MoO_3-Ag devices before and after electrical testing. (a) Optical micrograph of the Gr-MoO_3 heterostructure before electrode assembly (measuring position "P1"). (b) Optical micrograph of the post-test Gr-MoO_3 heterostructure after electrode disassembly (measuring positions "P2" and "P3"). (c) Comparison of the Raman spectra at the marked positions (positions "P1~P3").

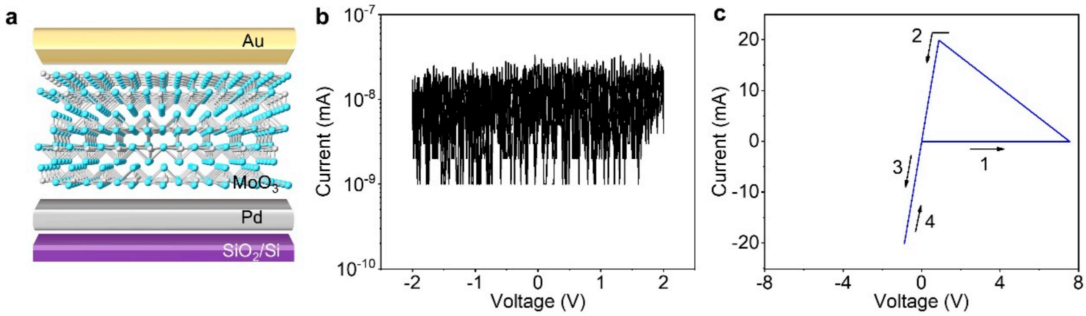

Figure S5. (a) Schematic diagram of the Pd-MoO_3-Au device (side view). (b) *I–V* characteristics in the range of −2 V to 2 V. (c) *I–V* characteristics in the range of −8 V to 8 V.

Table S3. Resistive switching performance, fabrication methods, and thickness optimization of the resistive layer in molybdenum oxide-based memristive devices.

Device structure	Set voltage	On/Off ratio	Retention	Resistance of HRS	Preparation method of molybdenum oxide	Thickness of molybdenum oxide	References
Mo/MoO_x/Ag	1.4 V	10^4	10^4 s	10^5 Ω	Straightforward oxidation of a Mo electrode in air	850 nm	[36]
Pt/MoO_x/ITO	2 V	90	10^4 s	10^4 Ω	Magnetron sputtering	66.5 nm	[37]
TiN/MoO_x/Cu	1 V	600	10^4 s	10^7 Ω	Reactive sputtering	50 nm	[38]
Pt/MoO_x/Ti	1 V	10^3	10^4 s	10^6 Ω	CVD	25 – 30 nm	[39]
FTO/MoO_3/Ag	2 V	500	10^3 s	4×10^5 Ω	CVD	500 nm	[40]
Pt/MoO_3/Ag<br>Gr/MoO_3/Ag	0.5 V<br>0.5 V	10^6<br>5×10^3	2×10^3 s<br>10^4 s	> 10^9 Ω<br>5×10^6 Ω	PVD and Mechanical exfoliation	18.3 nm<br>18.5 nm	This work

Table S4. Resistive switching characteristics, fabrication approaches, and switching layer thickness in 2D van der Waals material-based memristive devices.

Device structure	Set voltage	On/Off ratio	Retention	Preparation method of switch layer	Thickness of switch layer	References
Au/MoS_2/Ag	1 V	10^3	10^4 s	CVD	0.83 nm	[41]
Ag/h-BN/Ag	0.55 V	10^5	10^4 s	CVD	0.69 nm	[42]
Al/WS_2/ZnO/Ag	1.5 V	300	300 s	Sputtering	120 nm / 200 nm	[43]
(p+ Si)/WSe_2/Ag	1.5 V	10^5	---	PECVD	2 nm	[44]
Cu/h-BN/Ag	0.9 V	10^6	---	CVD	20 nm	[45]
Pt/MoO_3/Ag<br>Gr/MoO_3/Ag	0.5 V<br>0.5 V	10^6<br>5×10^3	2×10^3 s<br>10^4 s	PVD and Mechanical exfoliation	18.3 nm<br>18.5 nm	This work

Table S5. Resistive switching characteristics and number of resistance states in multi-level RRAM devices.

Device structure	Set voltage	On/Off ratio	Number of resistance states	References
Gr/α-In_2Se_3/h-BN/Cr/Au	4 V	10^9	32	[46]
Cr/Au/$HfSe_{2-x}O_x$/Cr/Au	3 V	10^5	7	[47]
Au/h-BN/Au	2.7 V	10^7	5	[48]
Pt/Ti_3C_2/Pt	4 V	5	9	[49]
Pt/MoO_3/Ag<br>Gr/MoO_3/Ag	0.5 V<br>0.5 V	10^6<br>5×10^3	8<br>9	This work

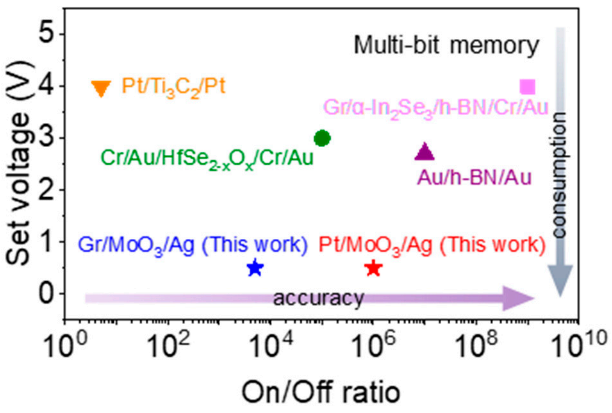

Figure S6. Comparison of the set voltage and On/Off ratio for different multi-level RRAM devices.
